# Quantitative Analysis of Diazepam Residues in Aquatic Products Using Magnetic Solid-Phase Extraction Combined with Ultra-High-Performance Liquid Chromatography–Tandem Mass Spectrometry

**DOI:** 10.3390/foods14234087

**Published:** 2025-11-28

**Authors:** Mengqiong Yang, Guangming Mei, Daoxiang Huang, Xiaojun Zhang, Pengfei He

**Affiliations:** 1 College of Food and Pharmaceutical Science, Zhejiang Ocean University, Zhoushan 316022, China; ymengqiong@163.com (M.Y.); huangdaoxiang2024@163.com (D.H.); 2 Zhejiang Marine Fisheries Research Institute, Zhoushan 316021, China; zhangxj@zjou.edu.cn (X.Z.); fuzhou.pengfei@zjou.edu.cn (P.H.)

**Keywords:** aquatic products, diazepam, magnetic solid-phase extraction, UPLC–MS/MS

## Abstract

A method combining magnetic solid-phase extraction (MSPE) with ultra-high performance liquid chromatography–tandem mass spectrometry (UPLC-MS/MS) was developed for the determination of diazepam residues in aquatic products. A novel magnetic nanoparticle material, Fe_3_O_4_@SiO_2_@DVB-NVP, was synthesized and applied as an adsorbent for sample cleanup. The sample preparation procedure involved extraction with 1% ammonia–acetonitrile, followed by purification using the MSPE technique to efficiently remove matrix interferents. Chromatographic separation was achieved on an ACQUITY UPLC BEH C_18_ column with a gradient elution program using a mobile phase composed of 0.1% formic acid–2 mM ammonium acetate solution and methanol. Detection was performed under multiple-reaction monitoring (MRM) mode with positive electrospray ionization (ESI^+^). Quantification was carried out using the external standard method. The synthesized magnetic material was characterized using SEM, TEM, FTIR, XRD, BET, and VSM, confirming its mesoporous structure, strong adsorption capacity, and excellent magnetic responsiveness. The method demonstrated good linearity over the concentration range of 0.25–50 μg/L (*r*^2^ = 0.997). The limits of detection and quantification were 0.20 μg/kg and 0.50 μg/kg, respectively. Average recoveries from spiked blank matrices at three levels (0.5, 2.5, and 5.0 μg/kg) ranged from 89.3% to 119.7%, with relative standard deviations (RSDs) between 0.8% and 10.2%. The proposed method is highly selective, exhibits minimal matrix interference, and provides reliable quantitative performance, making it suitable for the qualitative and quantitative analysis of diazepam residues in aquatic products.

## 1. Introduction

Diazepam is a benzodiazepine sedative–hypnotic drug exhibiting favorable pharmacological activities, such as hypnotic, anxiolytic, anticonvulsant, and antiepileptic activities [1,2,3]. It is widely used in clinical medicine [4]. However, diazepam residues in animal-derived food may be harmful to humans [5]. First, long-term intake of diazepam residues can lead to central nervous system depression in humans, posing significant health risks [6]. Second, diazepam residues can adversely affect the physiological functions of the animals themselves. Animal studies have shown that motor dysfunction, such as difficulties standing up, can occur when the plasma concentrations of diazepam in sheep reach 47.8–225 ng/mL [7,8]. In addition, diazepam residues can pose environmental risks by causing water pollution and affecting ecosystems. For example, diazepam has been detected in both source water (2.1 ng/L) and drinking water (23.5 ng/L) in the Marsden area of England [9]. In addition to its environmental persistence, diazepam exhibits a pronounced potential for bioaccumulation in aquatic organisms. A notable example is the common carp (*Carassius auratus*), which has been shown to have a bioconcentration factor (BCF) as high as 1238.6 [10]. This high BCF value indicates a significant risk: even when ambient water concentrations are at trace levels (ng/L), diazepam can be biomagnified through the food web, leading to potentially toxic concentrations in aquatic biota and posing a substantial ecological threat. Due to these risks, China has stipulated that diazepam is permitted only for therapeutic purposes in animal husbandry and should not be detectable in animal-derived food products [11]. However, the frequent occurrence of diazepam residues in freshwater products has been increasingly documented in recent years. For instance, a comprehensive monitoring study in Zhejiang Province, China, reported diazepam detection rates ranging from 0.33% to 4.67% during 2020–2024, with residue concentrations spanning from non-detectable levels up to 76.04 μg/kg and even reaching 112.61 mg/kg in contaminated bait sources [10]. Another nationwide survey also identified diazepam in 26% of sampled aquatic products, further confirming its non-negligible presence [12]. Critically, quantitative risk assessments based on monitoring data indicated that under the extreme dietary exposure scenarios, hazard quotients (HQs) for children approached the threshold of concern (HQ = 0.49) [10]. Therefore, these findings highlight the urgent need for rigorous quality and safety monitoring of diazepam residues in aquatic products to mitigate potential health risks. Given the diverse varieties and complex matrices of aquatic products, along with numerous interfering substances, it is particularly important to develop an accurate detection technology applicable to various typical aquatic product matrices (such as fish, crustaceans, and shellfish).

Current detection methods for diazepam analogs include enzyme-linked immunosorbent assay (ELISA) [13,14], high-performance liquid chromatography (HPLC) [15], gas chromatography–tandem mass spectrometry (GC-MS/MS) [16], and liquid chromatography–tandem mass spectrometry (HPLC-MS/MS) [17]. Among these methods, HPLC-MS/MS has become mainstream due to its high selectivity and sensitivity and accurate quantification ability, enabling simultaneous qualitative and quantitative analysis of multiple drug classes. Notably, sample pretreatment is crucial for ensuring assay reliability, and the choice of pretreatment technology directly impacts the method’s efficiency and cost-effectiveness. Current pretreatment methods for diazepam residue analysis include liquid–liquid extraction (LLE) [18], solid-phase extraction (SPE) [19], dispersive solid-phase extraction (DSPE) [20], molecularly imprinted solid-phase extraction (MISPE) [21], and magnetic solid-phase extraction (MSPE) [22]. As a third-generation green separation technology, MSPE offers significant advantages over traditional methods (such as LLE and SPE): (1) magnetic separation can replace centrifugation or filtration, dramatically shortening processing time and improving detection efficiency; (2) magnetic adsorbents are recoverable and reusable, thereby reducing reagent consumption and waste generation; and (3) reduced usage of organic reagents can greatly enhance environmental friendliness. MSPE can also be utilized in a wide range of applications and thus has considerable potential in the pretreatment of complex biological samples. However, conventional magnetic materials used have low adsorption selectivity and are susceptible to interference, thereby limiting the performance of MSPE in the detection of complex aquatic matrices. Therefore, developing functionalized magnetic adsorbents with high selectivity and anti-interference capacity is the key focus of current research. In this study, a method with reliable sensitivity and selectivity for detecting diazepam in aquatic products was developed using innovatively synthesized Fe_3_O_4_@SiO_2_@DVB-NVP magnetic composites combined with UPLC-MS/MS. The developed method provides a new technological solution for the monitoring and control of diazepam residues in aquatic products.

## 2. Materials and Methods

### 2.1. Chemicals and Reagents

Methanol, acetone, acetonitrile, and formic acid (chromatographically pure) were obtained from Merck KGaA (Darmstadt, Germany). Anhydrous ethanol, 2,2′-azobisisobutane (AIBN), MgSO_4_, 25% ammonia, ammonium acetate, sodium citrate, ethylene glycol, ferric chloride hexahydrate, sodium acetate, tetraethyl orthosilicate (TEOS), and 3-trimethoxysilylmethacrylatopropyl ester (MPS) (analytically pure) were purchased from Shanghai Sinopharm Chemical Reagent Co. (Shanghai, China). Divinylbenzene (DVB) and N-vinylpyrrolidone (NVP) were purchased from Shanghai Aladdin Industrial Co., Ltd. (Shanghai, China). Diazepam standard solution (methanol medium,1.0 mg/mL) was obtained from TMRM Quality Inspection Technology Co., Ltd. (Shanghai, China). Ultrapure water used in the experiments was prepared by a Milli-Q Advantage A10 system (Millipore, St. Charles, MO, USA).

### 2.2. Laboratory Instruments

UPLC-MS/MS analysis was performed using an Acquity UPLC I-Class system (Waters, Milford, MA, USA) coupled to a Xevo TQ-S triple quadrupole mass spectrometer equipped with an electrospray ionization (ESI) source. Sample pretreatment involved the following instruments: an MS3D vortex mixer (IKA, Staufen, Germany) for mixing, a DF-101S magnetic stirrer (Shanghai Bangxi Instrument Technology Co., Ltd., Shanghai, China) for stirring, an FJ200-SH high-speed homogenizer (Hangzhou SPARKLE Instruments Co., Ltd., Hangzhou, China) for homogenization, a model 5810 centrifuge (Eppendorf, Hamburg, Germany) for centrifugation, and an FS-2000T ultrasonic processor (Shanghai Sheng Analytical Ultrasonic Instrument Co., Ltd., Shanghai, China) for sonication.

The synthesized magnetic composites were characterized using a GeminiSEM 300 scanning electron microscope (Carl Zeiss AG, Baden-Württemberg, Germany), a Miniflex 600 X-ray diffractometer (Rigaku, Tokyo, Japan), and a Nicolet iS5 Fourier transform infrared (FTIR) spectrometer (Thermo Fisher, Waltham, MA,, USA). Magnetic properties were evaluated with a LakeShore 7404 vibrating sample magnetometer (LakeShore, Cleaveland, OH, USA), while specific surface area and porosity were determined using an ASAP 2460 automatic surface area and porosity analyzer (Micromeritics, Norcross, GA, USA).

### 2.3. Solution Preparation

Diazepam standard stock solution: the diazepam standard stock solution (10 μg/mL) was prepared by diluting 1.0 mL of the 1.0 mg/mL commercial standard solution to a final volume of 100 mL with methanol. The solution was stored at −20 °C in the dark.

Diazepam standard working solution: the diazepam standard working solution (100 ng/mL) was prepared by diluting 100 μL of the 1 mg/mL standard stock solution to a final volume of 10 mL with methanol. The solution was stored at −20 °C in the dark.

Ammonium acetate buffer solution: 7.7 g of ammonium acetate was dissolved in 480 mL of pure water, and the solution pH was then adjusted pH 5.2 with glacial acetic acid. Its volume was thereafter adjusted to 500 mL using pure water.

1% ammonia-acetonitrile solution: 40 mL of 25% ammonia was diluted with acetonitrile to a final volume of 1000 mL.

2 mmol/L ammonium acetate aqueous solution (containing 0.1% formic acid): 0.154 g of ammonium acetate was weighed and then dissolved in 950 mL of water. After 1 mL of formic acid was added, its final volume was adjusted to 1000 mL using water.

### 2.4. Experimental Methods

#### 2.4.1. Preparation of Fe_3_O_4_@SiO_2_@DVB-NVP

The preparation was carried out based on the method described in Liu et al. [23] with slight modifications. The procedure was as follows:

(1) Preparation of Fe_3_O_4_: 0.4 g of sodium citrate was dissolved in 72 mL of ethylene glycol in a 250 mL three-necked flask. Then, 16 mL of FeCl_3_·6H_2_O-ethylene glycol solution (5.0 g/100 mL) and 72 mL of sodium acetate-ethylene glycol solution (10 g/72 mL) were slowly added to the flask. The mixture was stirred vigorously at 1000 rpm for 30 min before being transferred to a 200 mL PTFE-lined stainless steel high-pressure reactor. The reactor was heated from room temperature to 180 °C at a rate of 5 °C/min and maintained at 180 °C for 10 h. After the reaction, the reactor was allowed to cool naturally to room temperature. The products were collected via magnetic separation, washed three times with deionized water and anhydrous ethanol, and then dried at 60 °C in a vacuum for 12 h to obtain Fe_3_O_4_ nanoparticles (NPS). The yield of Fe_3_O_4_ nanoparticles was 0.82 g (82% based on the mass of FeCl_3_·6H_2_O).

(2) Preparation of Fe_3_O_4_@SiO_2_: 1.0 g of Fe_3_O_4_ nanoparticles was dispersed in 300 mL of water initially, then ultrasonicated (40 kHz, 300 W) for 10 min to obtain a homogeneous suspension. The suspension was subsequently transferred to a 500 mL three-necked flask containing 100 mL of ethanol followed by the addition of 1.2 mL ammonia solution under nitrogen protection and magnetic stirring at 600 rpm, a mixture of 2.5 mL TEOS and 5.5 mL anhydrous ethanol was added dropwise. The reaction was then incubated in a water bath at 60 °C for 12 h. After completion of the reaction, the products were cooled naturally to room temperature, separated using a magnetic field, and washed three times with acetone and anhydrous ethanol, and then dried in a vacuum at 60 °C for 12 h, which yielded the final product, Fe_3_O_4_@SiO_2_ core-shell NPS. The yield of Fe_3_O_4_@SiO_2_ was 1.15 g (95% based on the initial mass of Fe_3_O_4_).

(3) Preparation of Fe_3_O_4_@MPS: 3.0 g of Fe_3_O_4_@SiO_2_ NPS was dispersed in 400 mL of an ethanol/H_2_O mixture (*v*/*v*, 3:1) and ultrasonicated (40 kHz, 300 W) for 10 min to ensure uniform dispersion. Under nitrogen protection at room temperature, 8 mL of ammonia was slowly added to the suspension, followed by dropwise addition of an MPS-ethanol solution (8 mL MPS dissolved in 10 mL ethanol) under constant magnetic stirring at 600 rpm. The reaction mixture was heated to 60 °C at a heating rate of 5 °C/min and continuously stirred for 12 h. At completion of the reaction, the products were cooled naturally to room temperature, separated using a magnetic field, and washed sequentially three times with pure water and anhydrous ethanol. The resulting Fe_3_O_4_@MPS composites were finally vacuum dried at 60 °C for 12 h and stored for subsequent use. The yield of Fe_3_O_4_@MPS was 3.21 g (93% based on the initial mass of Fe_3_O_4_@SiO_2_).

(4) Preparation of Fe_3_O_4_@SiO_2_@DVB-NVP: 3.0 g of Fe_3_O_4_@MPS was dispersed in 500 mL of acetonitrile and ultrasonicated (40 kHz, 300 W) for 10 min until uniformly dispersed. After that, 0.1 g of AIBN, 7.5 g of DVB, and 6.3 g of NVP were sequentially added to the dispersion. The mixture was magnetically stirred at 600 rpm for 30 min under nitrogen protection, heated to 75 °C at a heating rate of 5 °C/min, and then reacted under a nitrogen atmosphere for 16 h. Upon completion of the reaction, the magnetic NPS were cooled naturally to room temperature, separated using an external magnetic field, and washed three times with acetone and anhydrous alcohol. Finally, the washed magnetic NPS were dried under vacuum at 60 °C for 12 h to yield the final product. The yield of Fe_3_O_4_@SiO_2_@DVB-NVP was 5.77 g (192% based on the initial mass of Fe_3_O_4_@MPS).

#### 2.4.2. Structural Characterization of Magnetic NPS

Transmission electron microscopy (TEM), X-ray diffraction (XRD), scanning electron microscopy (SEM), vibrating sample magnetometer (VSM), FTIR, and Brunauer-Emmet-Teller (BET) were employed to characterize the physicochemical properties of the prepared magnetic nanoparticle samples. These include morphological features, crystal structure, surface functional groups, magnetic properties, and microporous structure [24].

For TEM analysis, 2.0 mg of sample was dispersed in anhydrous ethanol and sonicated for 10 min. A 4 μL aliquot of the resulting suspension was then deposited onto a copper mesh support film and allowed to dry naturally at room temperature. The prepared sample was examined under an accelerating voltage of 60 kV.

For SEM, sample was dispersed in anhydrous ethanol and sonicated for 10 min. A droplet of the suspension was placed on an aluminum sample stage pre-coated with a 5 nm Au film. Imaging was performed at an accelerating voltage ranging from 5 to 10 kV.

For XRD, the powdered sample was evenly spread at the center of a sample holder and scanned using Cu Kα radiation (λ = 0.15405 nm) across a 2θ range of 5° to 80°, with a scanning rate of 4°/min.

For FTIR, the sample was mixed with dried potassium bromide at a mass ratio of 1:100, thoroughly ground in an agate mortar to achieve homogeneity, and compressed into a transparent pellet. Spectral data were acquired in the range of 4000 to 400 cm^−1^ at a resolution of 4 cm^−1^, accumulated over 64 scans.

For VSM, the sample was wrapped with paraffin film, secured onto a vibrating sample holder, and analyzed at room temperature (300 K) under an applied magnetic field of ±20 kOe.

For BET, the samples were degassed at 120 °C for 6 h under a nitrogen atmosphere to remove substances on the surface. N_2_ adsorption-desorption isotherms were recorded at 77 K controlled using liquid nitrogen over a relative pressure range (P/P_0_) of 0.05–1.00.

#### 2.4.3. Sample Pretreatment

Aquatic product samples, including *Carassius auratus*, *Litopenaeus vannamei*, *Portunus trituberculatus*, and *Mytilus galloprovincialis*, were purchased from Fengmao Market, Lincheng, Zhoushan City, Zhejiang Province, China. Sampling and preparation were carried out according to GB/T 30891-2014 [25]. Homogenized samples were stored at −18 °C in a sealed storage container and thawed before use. For analysis, exactly 5.00 ± 0.01 g of the sample was weighed into a 50 mL polypropylene centrifuge tube. After adding 10 mL of 1% ammonia-acetonitrile extraction solution, the mixture was vortexed at 2500 rpm for 3 min. Subsequently, 4.0 g of anhydrous MgSO_4_ and 1.0 g of NaCl were introduced, followed by vortexing at 2500 rpm for another 3 min and centrifugation at 5000 rpm for 3 min. Next, 2.0 mL of the supernatant was transferred to a 10 mL centrifuge tube containing 40 mg of Fe_3_O_4_@SiO_2_@DVB-NVP adsorbent. The mixture was vortexed and shaken at 1000 rpm for 1 min followed by magnetic separation. Finally, 1.0 mL of the purified supernatant was filtered via a 0.22 μm organic phase membrane and transferred to a liquid phase injection bottle for analysis.

#### 2.4.4. Construction of Quantitative Standard Curves

Diazepam standard working solutions at various volumes (2.5, 5.0, 20, 50, 100, 200, and 500 μL) were accurately pipetted and diluted to 1.0 mL with mixture of 2 mmol/L aqueous ammonium acetate (containing 0.1% formic acid) and methanol (9:1, *v*/*v*). The resulting standard solutions had concentrations of 0.25, 0.5, 2, 5, 10, 20, and 50 μg/L, respectively. A calibration curve was constructed by plotting the mass spectral peak area on the vertical coordinate (*y*-axis) and the mass concentration on the horizontal coordinate (*x*-axis, μg/L).

#### 2.4.5. Chromatography-Mass Spectrometry Conditions

Chromatographic conditions were as follows: chromatographic column, ACQUITY UPLC^®^ BEH C18 column (2.1 × 100 mm, 1.7 μm); flow rate, 0.3 mL/min; column temperature, 30 °C; injection volume, 5 μL; mobile phase A, 2 mmol/L ammonium acetate containing 0.1% formic acid; and mobile phase B, methanol. The elution gradient was programmed as follows: 0.00–3.00 min, 10% B; 3.00–3.50 min, 15% B; 3.50–6.20 min, 68% B; 6.20–8.00 min, 95% B; and 8.00–10 min, 10% B.

Mass spectrometry conditions: ionization source, electrospray ionization in positive mode (ESI^+^); monitoring mode, multiple reaction monitoring (MRM, cone-well voltage = 36 V, quantitative and qualitative ion pairs = 285.1 > 154.1 and 285.1 > 193.1, respectively, and collision voltages = 30 V and 24 V, respectively); capillary voltage, 3.50 kV; ion source temperature, 120 °C; desolventization gas temperature, 380 °C; collision gas, high-purity argon; nitrogen flow rate in desolventization gas, 600 L/h; nitrogen flow rate in cone gas, 50 L/h.

#### 2.4.6. Evaluation of Matrix Effects

During mass spectrometry, coextracted matrix components such as proteins, polysaccharides, and lipids might interfere with the target’s ionization, resulting in either signal suppression or enhancement, collectively known as matrix effect (ME). In the present study, matrix effects were evaluated by comparing the slope of the matrix-matched standard curve with that of the solvent-based calibration curve, using the formula: ME = (slope of matrix-matched curve/slope of solvent-based curve − 1) × 100%. Positive ME values denote a matrix-enhancing effect, while negative values indicate a matrix-inhibitory effect. According to the established thresholds, ∣ME∣ ≤ 20% indicates a weak matrix effect, 20% < ∣ME∣ ≤ 50% indicates a moderate matrix effect, and ∣ME∣ > 50% indicates a strong matrix effect [26].

#### 2.4.7. Methodological Sensitivity and Accuracy

After sample pretreatment and online analysis (as described in Section 2.4.3 and Section 2.4.5), the sensitivity of the method was investigated using mass spectrometry. The limit of detection (LOD) was determined based on a signal-to-noise ratio (S/N) of 3, and the limit of quantification (LOQ) was calculated using an S/N of 10.

The accuracy of the method was investigated by spiking diazepam standard solution into different blank matrix samples of *Larimichthys crocea*, *Trachypenaeus curvirostris*, *Portunus trituberculatus*, *Carassius auratus*, and *Mytilus galloprovincialis* at concentrations of 0.5, 2.5, and 5.0 μg/kg, respectively. After spiking, the samples were processed according to the described method, and the measured values were compared with the theoretical values. The recovery, which reflects the accuracy of the method, was calculated as the ratio of the measured value to the spiked value. The relative standard deviation (RSD) of parallel determinations was employed to evaluate the method’s reproducibility.

### 2.5. Data Analysis

Data were analyzed using analysis of variance (ANOVA) and a Least Significant Difference (LSD) test. All analyses were performed using Excel 2010, with results considered statistically significant at *p* < 0.05. Graphs were created using Origin 2025, and results were expressed as mean ± standard deviation.

## 3. Results and Discussion

### 3.1. Material Characterization

#### 3.1.1. TEM

**Figure 1:**
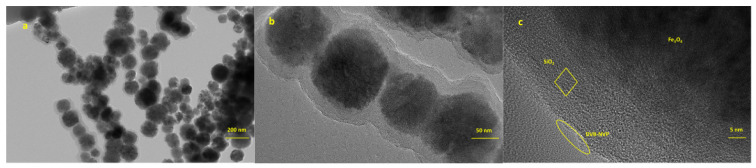
TEM images of Fe_3_O_4_@SiO_2_@DVB-NVP with different sizes: 200 nm (**a**), 50 nm (**b**), and 5 nm (**c**).

TEM was used to investigate the microscopic morphology and structural features of Fe_3_O_4_@SiO_2_@DVB-NVP composites. As shown in Figure 1a, the Fe_3_O_4_@SiO_2_@DVB-NVP particles were uniformly distributed with good dispersion. High-resolution TEM images (Figure 1b,c) showed clear lattice fringes of Fe_3_O_4_ and an interfacial amorphous SiO_2_ layer, confirming that the Fe_3_O_4_ nanoparticles were completely encapsulated by SiO_2_. The outermost layer had a rough coating due to the DVB-NVP copolymer shell.

#### 3.1.2. SEM

**Figure 2:**
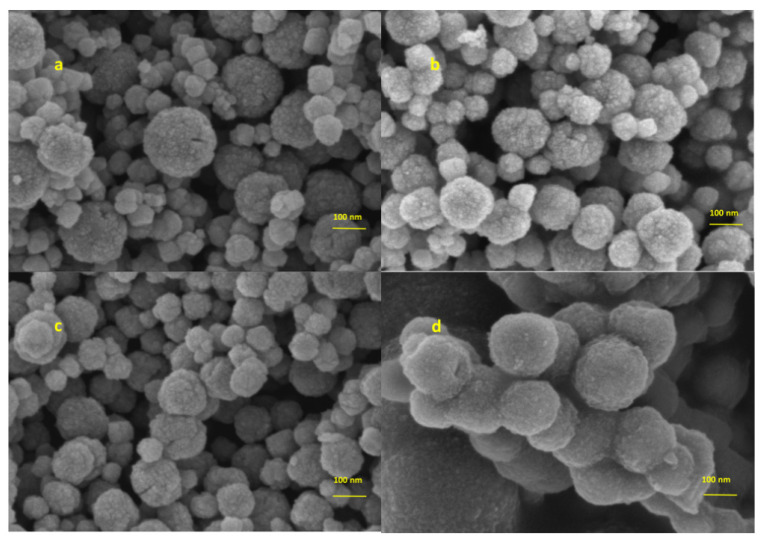
SEM images of (**a**) Fe_3_O_4_, (**b**) Fe_3_O_4_@SiO_2_, (**c**) Fe_3_O_4_@MPS, and (**d**) Fe_3_O_4_@SiO_2_@DVB-NVP.

SEM was used to characterize the morphology of Fe_3_O_4_, Fe_3_O_4_@SiO_2_, Fe_3_O_4_@MPS, and Fe_3_O_4_@SiO_2_@DVB-NVP nanoparticles. As shown in Figure 2a, Fe_3_O_4_ particles exhibited a regular spherical morphology with an average particle size of ~180 nm. However, some agglomeration was observed between the particles, likely due to magnetic dipole interactions and van der Waals forces. In Figure 2b, the Fe_3_O_4_@SiO_2_ particles had a smooth core-shell structure, indicating that SiO_2_ was uniformly coated on the surface of Fe_3_O_4_ through ammonia-catalyzed hydrolytic condensation reaction. After surface modification with MPS, a rougher surface was observed due to condensation between the silanol group (Si-OH) and MPS, which introduced double-bonded functional groups (Figure 2c). Finally, a free radical copolymerization reaction of divinylbenzene (DVB) and N-vinylpyrrolidone (NVP) was initiated by AIBN, resulting in the formation of a polymer capping layer on the Fe_3_O_4_@SiO_2_ surface (Figure 2d). The average particle size of Fe_3_O_4_@SiO_2_@DVB-NVP composites increased to ~202 nm, indicating successful polymer encapsulation.

#### 3.1.3. XRD

**Figure 3:**
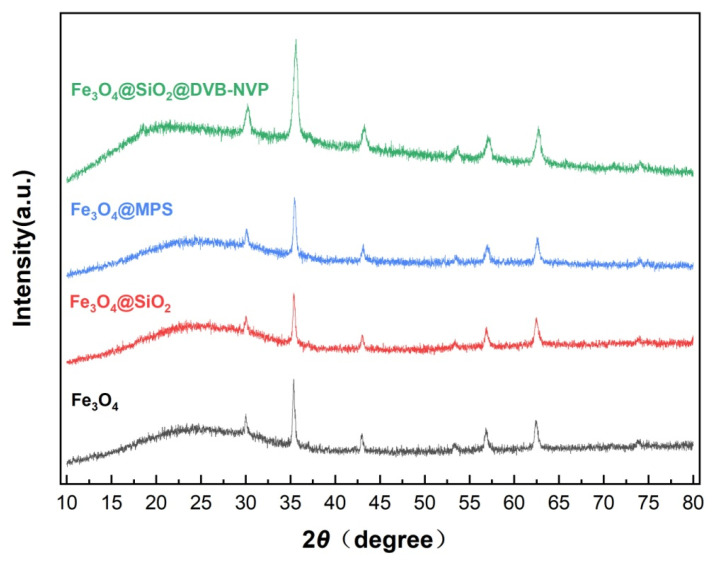
XRD images of Fe_3_O_4_, Fe_3_O_4_@SiO_2_, Fe_3_O_4_@MPS, and Fe_3_O_4_@SiO_2_@DVB-NVP.

Figure 3 illustrates the XRD spectra of Fe_3_O_4_, Fe_3_O_4_@SiO_2_, Fe_3_O_4_@MPS, and Fe_3_O_4_@SiO_2_@DVB-NVP nanoparticles. The XRD spectra of Fe_3_O_4_ showed diffraction peaks at 2θ = 30.1°, 35.6°, 43.3°, 53.9°, and 62.7°, corresponding to the (220), (311), (400), (422), and (440) planes, respectively. The planes are consistent with the standard card of magnetite (JCPDS No. 96-900-9769), confirming the cubic spinel structure of Fe_3_O_4_ [27]. Among these peaks, the intensity and sharpness of the peak at 2θ = 35.6° indicate high crystallinity of Fe_3_O_4_. For Fe_3_O_4_@SiO_2_, a broad, weak amorphous “bun peak”, which is a typical amorphous SiO_2_ peak, was observed between 20° and 30°. The XRD spectra of Fe_3_O_4_@SiO_2_, Fe_3_O_4_@MPS, and Fe_3_O_4_@SiO_2_@DVB-NVP were similar to that of Fe_3_O_4_, and no new peaks were observed, indicating that the SiO_2_ coating, MPS-modified, and DVB-NVP polymer layers were amorphous [28]. Importantly, the characteristic diffraction peaks of Fe_3_O_4_ were intact in all composites, suggesting that the SiO_2_ coating, along with the subsequent polymer modification process, has no impact on the crystal structure of Fe_3_O_4_.

#### 3.1.4. FTIR

**Figure 4:**
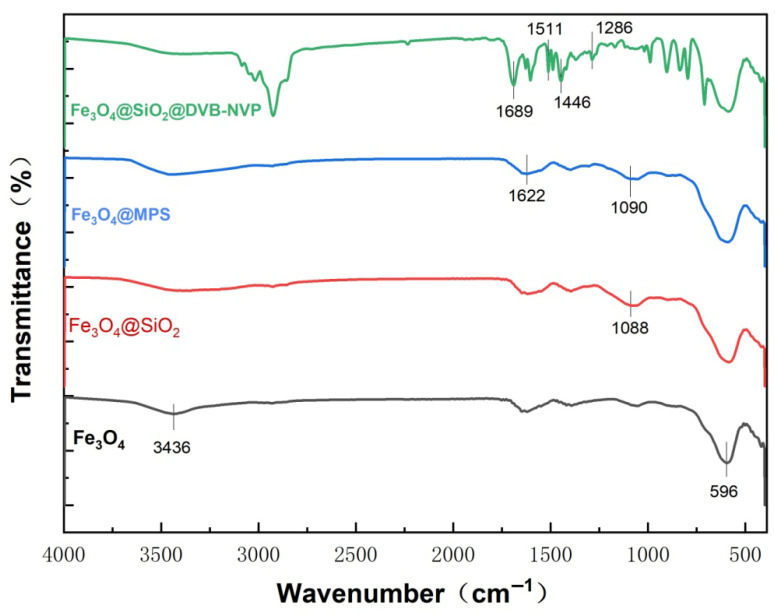
FTIR images of Fe_3_O_4_, Fe_3_O_4_@SiO_2_, Fe_3_O_4_@MPS, and Fe_3_O_4_@SiO_2_@DVB-NVP.

The surface functional groups of the material were systematically characterized using FTIR (Figure 4). The broad peak at 3436 cm^−1^ in Fe_3_O_4_ is attributed to the stretching vibration of the surface O-H group, and the characteristic peak at 596 cm^−1^ corresponds to the Fe-O stretching vibration of the spinel Fe_3_O_4_. Compared to pristine Fe_3_O_4_, the Si-O-Si antisymmetric stretching vibration peak at 1088 cm^−1^ of Fe_3_O_4_@SiO_2_ was more intense, confirming that SiO_2_ was successfully coated on the Fe_3_O_4_ surface. After modification with MPS, the vibrational peak of Si-O-Si in Fe_3_O_4_@MPS was shifted to 1099 cm^−1^, indicating that the condensation reaction between MPS and SiO_2_ surface generated a new Si-O bond. Meanwhile, the characteristic C=C bond in MPS appeared at 1622 cm^−1^, which proves that MPS was successfully grafted onto the surface of SiO_2_ through siloxane bonding (Si-O-Si), and polymerizable double-bonding functional groups were introduced. The IR spectrum of Fe_3_O_4_@SiO_2_@DVB-NVP showed a peak corresponding to the C-N stretching vibration of the pyrrolidinone ring in NVP at 1286 cm^−1^, a peak for the C=O stretching vibration at 1689 cm^−1^, and a peak for the DVB benzene ring backbone at 1511 cm^−1^. The spectrum also showed a peak corresponding to the C-H stretching vibration at 2924 cm^−1^, which mainly originates from the methylene group (-CH_2_-) of DVB and the aliphatic group of the alkyl chain of NVP. The disappearance of the C=C peak at 1622 cm^−1^ of Fe_3_O_4_@SiO_2_@DVB-NVP indicates the involvement of the double bond in the polymerization reaction, confirming the successful copolymerization of DVB with NVP, which generates a cross-linked polymer shell layer.

#### 3.1.5. VSM

**Figure 5:**
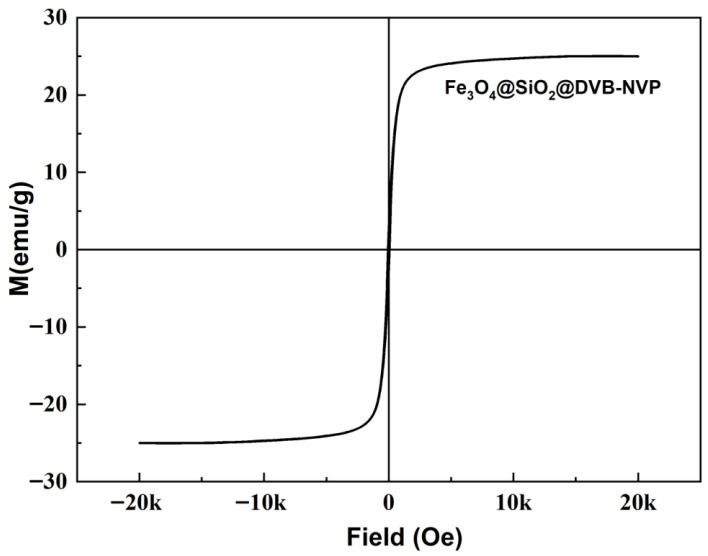
VSM image of Fe_3_O_4_@SiO_2_@DVB-NVP.

The magnetic properties of the prepared materials were characterized using VSM. As shown in Figure 5, the saturation magnetization strength (*Ms*) of Fe_3_O_4_@SiO_2_@ DVB-NVP was 25.01 emu/g, and the coercivity (*Hc*) was close to zero. This finding indicates that Fe_3_O_4_@SiO_2_@ DVB-NVP nanoparticles exhibit the characteristics of soft magnetic materials.

#### 3.1.6. BET

**Figure 6:**
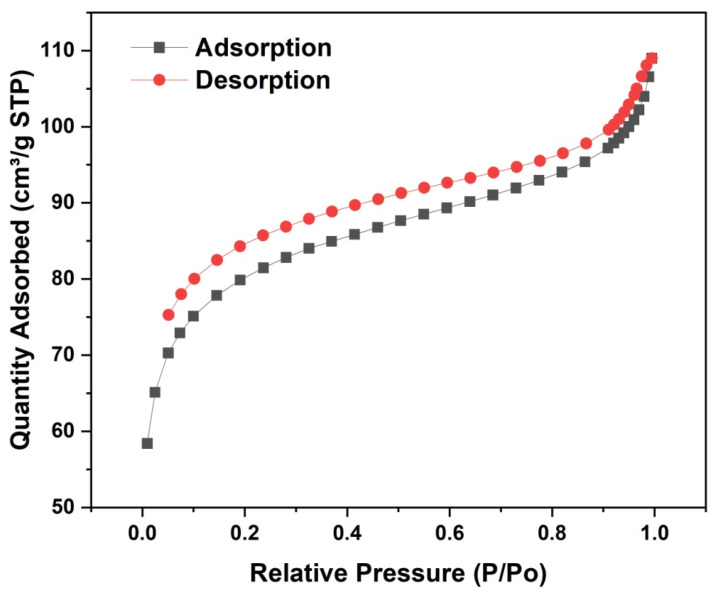
Nitrogen adsorption-desorption isotherm curve of Fe_3_O_4_@SiO_2_@DVB-NVP.

The mesoporous structural properties of Fe_3_O_4_@SiO_2_@DVB-NVP NPS were characterized based on the specific surface area and pore size distribution via the nitrogen adsorption-desorption method. As shown in Figure 6, the material’s BET-specific surface area was 300.21 m^2^/g, with a single-point adsorption total pore volume of 0.168 cm^3^/g. The average pore diameter, calculated using the BJH method, was 4.66 nm for adsorption and 4.54 nm for desorption. The nitrogen adsorption-desorption isotherm conformed to the type IV isotherm (IUPAC classification). At low relative pressures (0 < P/P_0_ < 0.1), the adsorption rate increases rapidly with the increase in pressure, indicating that the adsorption is monolayer. As the relative pressure was further increased, the adsorption gradually transitioned to multilayer adsorption. When the relative pressure reached at P/P_0_ = 0.4–0.8, the adsorption-desorption curves showed an obvious H1-type hysteresis loop, which is characteristic of materials with a highly ordered mesoporous structure and adsorption behavior following the capillary coalescence mechanism. However, the isotherms were not completely closed in the low-pressure region, probably due to the dissolution or structural deformation of the flexible polymer component (DVB-NVP) during high-pressure adsorption, which could not be completely recovered during the desorption process.

### 3.2. Optimization of Sample Pretreatment for MSPE

#### 3.2.1. Optimization of Extraction Solvent Composition

**Figure 7:**
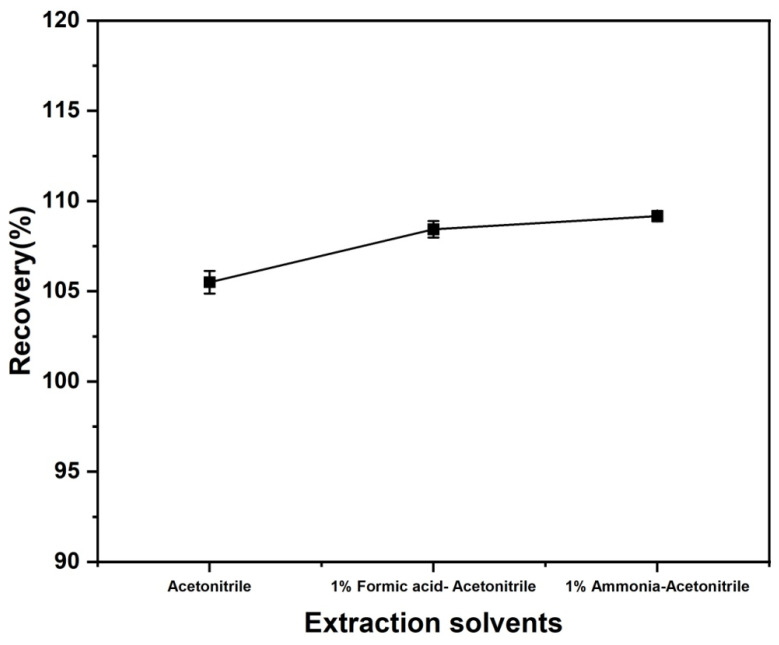
Effect of extraction solvent composition on the recovery of diazepam spiked into *Carassius auratus* samples.

To optimize the efficiency in the extraction of diazepam residues in aquatic products, three extraction solvents were evaluated: pure acetonitrile, 1% formic acid-acetonitrile, and 1% ammonia-acetonitrile. The extraction performance of each solvent was assessed using blank *Carassius auratus* samples spiked with diazepam at a concentration of 2.0 μg/kg. As illustrated in Figure 7, 1% ammonia-acetonitrile had superior extraction efficiency, yielding higher diazepam recoveries compared to the other solvents, in addition to a lower relative standard deviation (RSD). Diazepam, which is a weakly basic drug (pKa ~ 3.3), remained predominantly non-dissociated neutral molecules form under alkaline conditions (pH ~ 9–10) due to the presence of 1% ammonia-acetonitrile. According to the “like dissolves like” principle, the non-dissociated form of diazepam is more hydrophobic and thus has a higher partition coefficient in acetonitrile. For this reason, its desorption from the sample matrix was enhanced. Acidic acetonitrile, as an efficient protein precipitant, can rapidly denature and coagulate proteins in the sample extract, thereby releasing diazepam entrapped or adsorbed within the protein network. This may explain why the extraction efficiency of 1% formic acid-acetonitrile was superior to that of pure acetonitrile. Based on these findings, 1% ammonia-acetonitrile was selected as the optimal extraction solvent for subsequent experiments.

#### 3.2.2. Optimization of Extraction Solvent Volume

**Figure 8:**
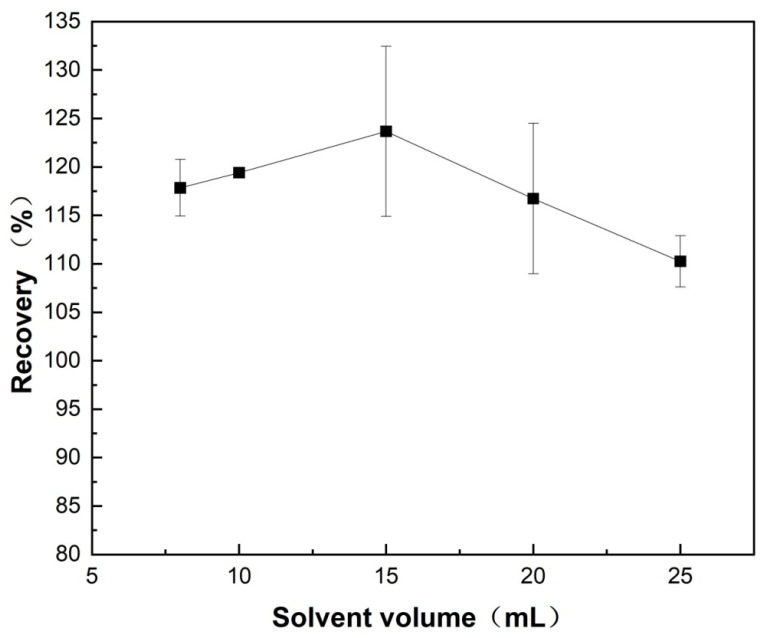
Effect of extraction solvent volume on the recovery of diazepam spiked into *Carassius auratus* samples.

The effect of the volume of extraction solvent on the extraction efficiency was systematically optimized. The extraction using 1% ammonia-acetonitrile in five gradient volumes (8, 10, 15, 20, and 25 mL) was investigated (Figure 8). The results showed that the recovery of the 15-mL group was abnormally high (123.6%), while the reproducibility was poor (RSD = 15.4%). For the 8-mL group, the volume of supernatant obtained after treatment with anhydrous MgSO_4_ and NaCl was insufficient for further analysis. The average recoveries for the 10-, 20-, and 25-mL groups were 118.5%, 116.5%, and 110.5%, respectively. The difference between these groups was not statistically significant (*p* > 0.05). Considering green chemistry principles (i.e., reducing organic solvents use), method stability, and practical handling, 10 mL (RSD = 0.86%) was selected as the optimal extraction volume.

#### 3.2.3. Optimization of Anhydrous MgSO_4_ Amount

**Figure 9:**
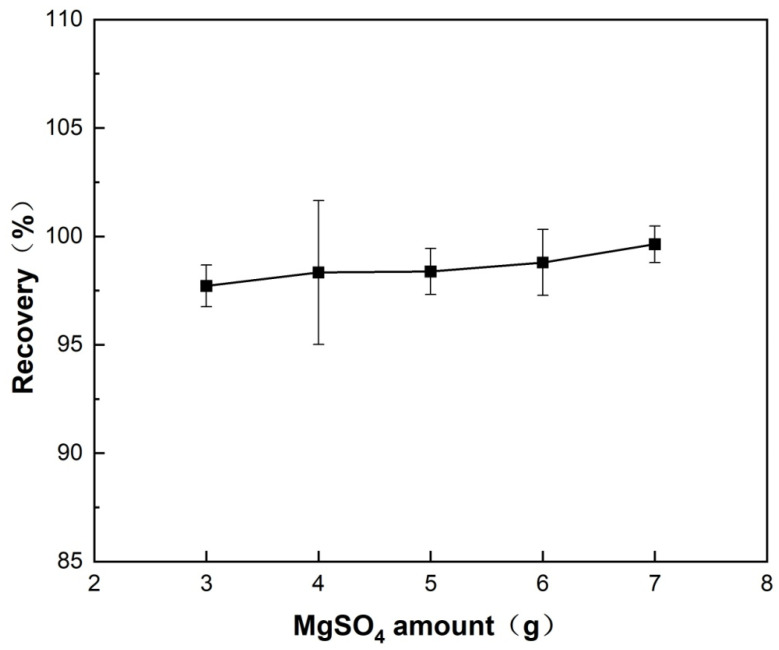
Effect of anhydrous MgSO_4_ amount on the recovery of diazepam spiked into *Carassius auratus* samples.

The effect of varying anhydrous MgSO_4_ amounts (3–8 g) with a fixed MgSO_4_:NaCl mass ratio (4:1) on the recovery of diazepam was systematically investigated. As shown in Figure 9, the recovery increased with the increase in MgSO_4_ dosage. The enhancement may be attributed to two main mechanism: (1) anhydrous MgSO_4_ removes free water from the extraction solution due to its strong water absorption capacity and thereby improves the purity of the organic phase (acetonitrile), and (2) it synergizes with the salting-out effect of NaCl, reducing the solubility of the target in the aqueous phase and facilitating its partitioning to the acetonitrile phase. Although the recoveries across the 3–7 g range were relatively high and did not show statistically significant differences (*p* > 0.05), the selection of the optimal MgSO_4_ amount was based not only on recovery but also on practical and mechanistic considerations. Specifically, when the amount of MgSO_4_ was less than 4 g, the system showed signs of incomplete dehydration, as indicated by the presence of residual water in the acetonitrile phase. This residual moisture can compromise the porosity and accessibility of the magnetic adsorbent in subsequent steps, potentially leading to reduced extraction efficiency and reproducibility. On the other hand, when the amount of MgSO_4_ exceeded 7 g, the mixture became excessively solidified, which significantly reduced the volume of the supernatant available for the following magnetic solid-phase extraction step, thereby impairing operational feasibility. Therefore, considering both the high recovery (98.5% at 4 g) and the need for a balance between complete dehydration and maintaining sufficient supernatant volume, 4 g of anhydrous MgSO_4_ was selected as the optimal amount for subsequent experiments.

#### 3.2.4. Optimization of Fe_3_O_4_@SiO_2_@DVB-NVP Amount

**Figure 10:**
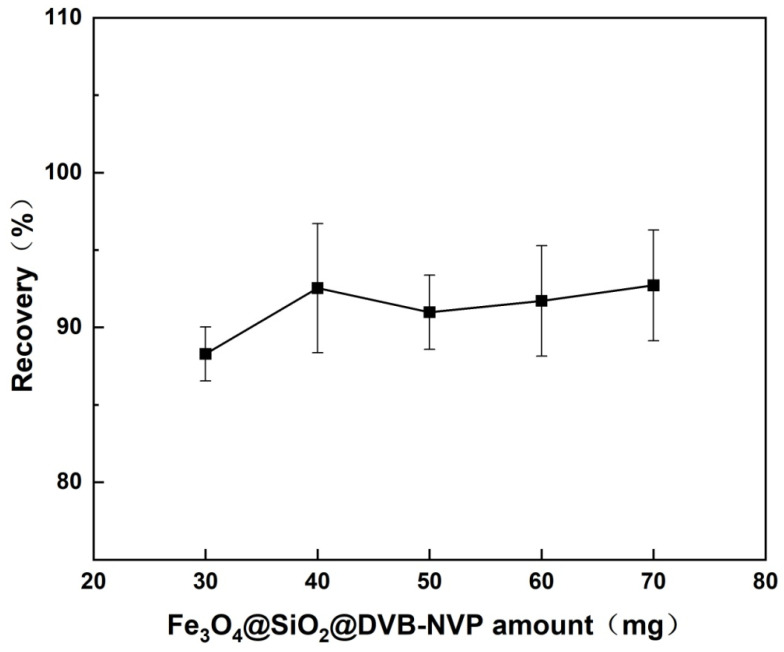
Effect of adsorbent amount on the recovery of diazepam spiked into *Carassius auratus* samples.

The influence of Fe_3_O_4_@SiO_2_@DVB-NVP amount (30–70 mg) on the recovery of diazepam was systematically investigated. As shown in Figure 10, the highest recovery (92.8%) was achieved at an adsorbent amount of 40 mg. When the amount was reduced to 30 mg, the recovery decreased to 88.5%, likely due to insufficient adsorption sites and lower impurity removal efficiency. Notably, the recovery was only 58.6% when no adsorbent was used, clearly demonstrating the key role of the adsorbent in eliminating matrix interference. When the amount was increased to 50–70 mg, the recovery did not significantly enhance (*p* > 0.05). This outcome may be attributed to: (1) particle agglomeration, which can reduce the effective specific surface area, and (2) secondary adsorption effects, which may cause loss of target analyte. The 40-mg dosage not only led to optimal extraction efficiency but also offered a significant operational advantage by shortening the magnetic separation time from 2–3 min (in the conventional method) to 30 s [29]. This finding confirms the efficacy of the magnetic solid-phase extraction technique in selectively eliminating matrix interference. Based on the comprehensive performance evaluation, 40 mg was considered the optimal adsorbent dosage.

### 3.3. Matrix Effects

**Figure 11:**
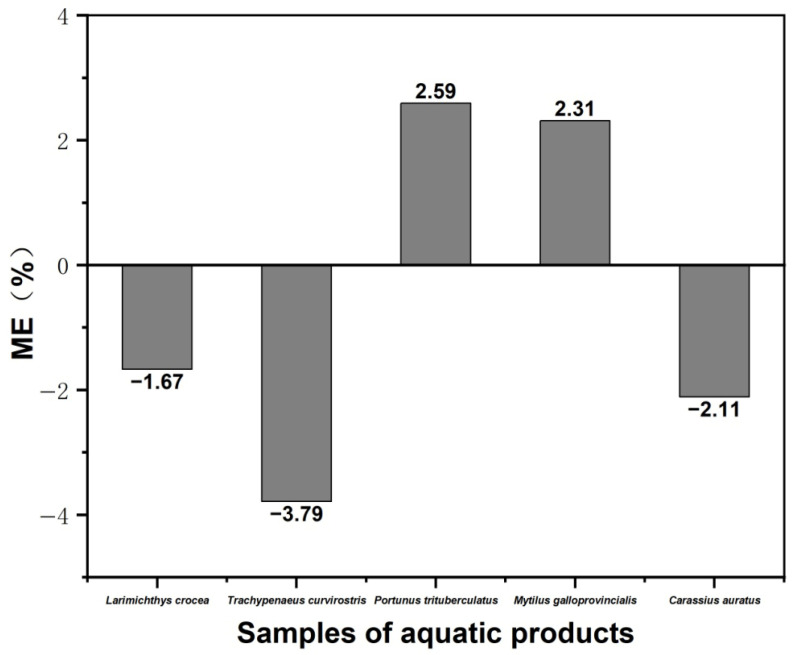
Matrix effects in different aquatic products on diazepam quantification.

Matrix effects arise primarily from incomplete purification during sample pretreatment and ionization competition during mass spectrometric detection. If matrix components are not effectively removed, they may interfere with the ionization efficiency of the target analyte, resulting in signal enhancement or suppression. These effects can compromise the accuracy of quantitative results. To assess matrix effects, the slope ratio between the matrix-matched calibration curve and the solvent-based calibration curve was calculated. As illustrated in Figure 11, matrix suppression was observed in *Larimichthys crocea*, *Trachypenaeus curvirostris* and *Carassius auratus*, while matrix enhancement was observed in *Portunus trituberculatus* and *Mytilus galloprovincialis*. The degree of the matrix effect of diazepam in five aquatic products ranged from −3.79% to 2.59%. This is considered a weak matrix effect. The results demonstrate that the sample pretreatment process can effectively reduce matrix interferences, which allows for the direct quantification using the solvent-based calibration curve.

### 3.4. Standard Curve for Diazepam

**Figure 12:**
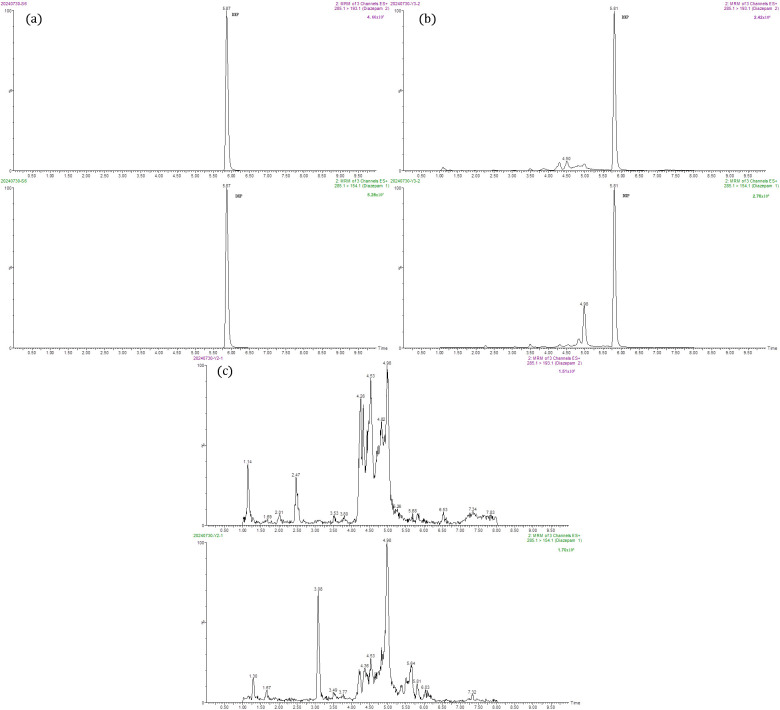
MRM chromatograms of diazepam in (**a**) a 50 μg/L standard solution, (**b**) a diazepam-positive crucian carp sample and (**c**) a negative control crucian carp sample.

In the concentration range of 0.25–50 μg/L, the linear calibration curve for diazepam was established between the peak area (*y*) obtained from mass spectrometry and the mass concentration (*x*, μg/L), described by the equation *y* = 1.0109*x* + 0.1281. The correlation coefficient (*r*^2^) reached 0.9970, demonstrating a good linear relationship. Under the optimized chromatographic-mass spectrometry conditions, the MRM chromatogram of a 50 μg/L diazepam standard solution was obtained as shown in Figure 12a. Figure 12b,c displayed the MRM chromatograms of a negative control sample from crucian carp and a diazepam-positive sample from the same species, respectively. The results indicated that diazepam eluted at approximately 5.8 min, exhibiting a sharp and symmetric peak. No significant interfering peaks were observed at the corresponding retention time, which further confirmed the effectiveness of the sample pretreatment method in removing matrix interference.

### 3.5. Method Evaluation

#### 3.5.1. Method Sensitivity

The limit of detection (LOD) and limit of quantification (LOQ) were defined as the concentrations yielding signal-to-noise (S/N) ratios of 3 and 10, respectively, based on chromatographic peaks from blank spiked samples. The results showed that the LOD for diazepam in all five samples (*Larimichthys crocea*, *Trachypenaeus curvirostris*, *Carassius auratus*, *Portunus trituberculatus* and *Mytilus galloprovincialis*) was 0.20 μg/kg, and the LOQ was 0.50 μg/kg.

#### 3.5.2. Method Accuracy and Precision

Blank matrix samples of five representative aquatic products were spiked with diazepam at concentrations of 0.5, 2.5, and 5 μg/kg. Each spiked level was tested in six replicate samples, and the mean recovery and relative standard deviation (RSD, *n* = 6) were calculated. As shown in Table 1, average recoveries ranged from 89.3% to 119.7%, with RSDs between 0.8% and 10.2%. Although some recoveries were slightly elevated (up to 119.7%), all values fell within the acceptable range of 70–120%. This systematic positive bias may stem from the adsorbent’s high purification efficiency or undetected matrix components. Given the low RSDs (≤10.2%) and consistent performance across diverse matrices, the method has reliable quantitative performance and is suitable for the analysis of diazepam residues in various aquatic products, including fish, crustaceans, and shellfish.

As shown in Table 2, the present method was systematically compared with existing approaches for determining diazepam residues in aquatic products, including the standard Chinese method SN/T 3235-2012 [30]. The comparative analysis revealed that while the present method exhibited a slightly higher LOD/LOQ (0.20/0.50 μg/kg) than some advanced methods employing matrix-matched calibration with internal standard correction, it demonstrated clear and practical advantages in processing efficiency, cost-effectiveness, and green chemistry compatibility. These merits were highlighted below: (1) Improved sample purification via MSPE: unlike the conventional QuEChERS or SPE/LLE methods, this method utilized a novel Fe_3_O_4_@SiO_2_@DVB-NVP magnetic adsorbent. Its mesoporous structure (pore size ~4.6 nm) and high surface area (300.21 m^2^/g) enabled effective removal of matrix interferents, while its magnetic properties (saturation magnetization 25.01 emu/g) allowed rapid separation within 30 s. This simplified pretreatment by omitting steps such as elution and nitrogen blowing, reducing the total time to under 30 min; (2) Reduced matrix effects and operational complexity: whereas many existing methods required matrix-matched calibration with internal standards to compensate for significant matrix effects, the present method achieved negligible interference (|ME| ≤ 3.79%) across various sample types. This performance permitted the use of a solvent-based external standard curve, thereby lowering costs and simplifying operations without compromising quantitative reliability. The observed recovery range (89.3–119.7%), although slightly elevated in some cases, remained within the acceptable range and could be attributed to the adsorbent’s high purification efficiency, which reduces matrix suppression. All recoveries showed satisfactory precision (RSDs ≤ 10.2%), supporting the method’s robustness; (3) Green chemistry advantages and sufficient sensitivity: the method used less organic solvent (e.g., 10 mL per extraction) and achieved an LOQ (0.50 μg/kg) fully adequate for routine monitoring.

Overall, the present method provided a more efficient, economical, and reliable alternative for routine monitoring of diazepam in aquatic products compared to existing techniques.

### 3.6. Application to Authentic Samples

Using the proposed method, a targeted surveillance of diazepam residues was carried out on 343 samples of farmed freshwater aquatic products, which were collected from representative aquaculture regions distributed across 11 prefecture-level cities in Zhejiang Province. The sampled aquatic products comprised farmed fish (274 samples), farmed shrimp (24 samples), farmed crabs (21 samples), and farmed turtles (24 samples). Test results indicated that diazepam was detected in 37 samples, all of which were farmed fish, whereas no diazepam was detected in samples of farmed shrimp, crabs, or turtles (all below the LOD of 0.5 μg/kg). The overall pass rate of the monitored samples stood at 89.2%, with the pass rate for farmed fish alone reaching 86.5%. Details regarding the detection of diazepam in positive samples are presented in Table 3, with residue concentrations ranging from 0.52 to 55.65 μg/kg. The positive samples involved multiple fish species, including *Carassius auratus*, *Cyprinus carpio*, *Mylopharyngodon piceus*, *Channa argus*, and *Opsariichthys bidens*. The analysis of practical samples demonstrated the method’s effectiveness for the trace detection of diazepam in complex aquatic product matrices.

## 4. Conclusions

In this study, a sensitive and efficient method was established for determining diazepam residue concentrations in aquatic products by combining MSPE with Fe_3_O_4_@SiO_2_@DVB-NVP and UPLC-MS/MS. The synthesized adsorbent displayed strong magnetic responsiveness (saturation magnetization: 25.01 emu/g), a high specific surface area (300.21 m^2^/g), and notable selectivity, enabling effective purification of complex matrices. The method exhibited a wide linear range (0.25–50 μg/L, *r*^2^ = 0.997), low detection and quantification limits (0.20 and 0.50 μg/kg, respectively), and satisfactory accuracy with recoveries of 89.3–119.7%. It also minimized organic solvent use, shortened processing time, and showed negligible matrix effects (|ME| ≤ 3.79%), allowing quantification with a solvent-based calibration curve. Successful application to real aquatic samples confirmed its practicality.

Notwithstanding these advantages, this method relies on a lab-synthesized adsorbent, which presents challenges in batch-to-batch reproducibility and potential analyte loss during adsorption, limiting its current commercial availability compared to conventional SPE cartridges. Future work should prioritize standardizing and scaling up the adsorbent synthesis, systematically investigating adsorption/desorption dynamics to minimize analyte loss, and assessing commercial feasibility to facilitate technology transfer from laboratory to routine application. Further efforts should also expand the method’s applicability to other benzodiazepines and high-lipid matrices, supporting its broader role in food and environmental safety monitoring.

## Figures and Tables

**Table 1:** Spike recovery and precision of diazepam in five aquatic product matrices (*n* = 6).

Matrix	Added Level (μg/kg)					
	0.5		2.5		5.0	
	Recovery (%)	RSD (%)	Recovery (%)	RSD (%)	Recovery (%)	RSD (%)
*Larimichthys crocea*	119.7	0.8	117.4	3.7	119.6	1.3
*Trachypenaeus curvirostris*	119.1	3.2	113.2	5.6	115.9	4.8
*Portunus trituberculatus*	99.7	7.4	89.3	3.2	103.2	2.5
*Mytilus galloprovincialis*	117.2	6.4	111.3	10.2	115.9	6.1
*Carassius auratus*	114.5	2.4	115.8	3.2	114.7	1.4

**Table 2:** Method comparison for diazepam analysis in aquatic products.

Instrument	Matrix	Purification	Quantification	LOD (μg/kg)	LOQ (μg/kg)	Linear Range (μg/L)	Spiked Recovery (%)	RSD (%)	Literature
LC-MS/MS	Fish	QuEChERS (PSA, C18)	Matrix-matched calibration curve with internal standard correction	/	0.50	/	76.5–108	/	[30]
UPLC-MS/MS	Carps, *Hypophthalmichthys nobilis*, grass carp, tilapia, catfish, crucian carp, turbot, shrimp, mussel, *Hypophthalmichthys molitrix*	n-hexane LLE & SPE (Florisil, C18, PSA, NH_2_)	Matrix-matched calibration curve with internal standard correction	0.03–0.08	0.10–0.24	0.1–100	81.6–113	0.9–7.5	[31]
LC-MS/MS	Fish and shrimp	C_18_ SPE	Matrix-matched calibration curve	0.01	0.03	0.05–40	77.94–104.27	1.19–4.76	[32]
LC-MS/MS	*Anguilla anguilla*	HLB Oasis SPE and Captiva EMR-lipid SPE	Matrix-matched calibration curve with internal standard correction	0.22	0.75	0–100	82	6.00	[33]
HPLC-ESI-MS/MS	Carp	QuEChERS (PSA)	Matrix-matched calibration curve	0.50	2.50	2.50–100	96–108.8	4.5–5.5	[34]
UHPLC–MS/MS	*Carassius auratus*	n-hexane. LLE	Matrix-matched calibration curve with internal standard correction	0.10	0.30	0.3–100	92.2–103.2	<7.83	[35]
UPLC-MS/MS	Freshwater fish	QuEChERS (C18)	Matrix-matched calibration curve with internal standard correction	0.53	1.76	0.1–50.0	96.1–97.6	4.3–6.2	[36]
UPLC-Q Exactive MS	*Carassius auratus*, *Macrobrachium nipponense*	Turboflow online SPE	Matrix-matched calibration curve with internal standard correction	0.50	2.00	1.0–100.0	75.3–110.0	<10	[37]
UPLC-MS/MS	Grass carp, tilapia, crucian, silver carp, bighead carp	QuEChERS (Florisil, C18)	Matrix-matched calibration curve with internal standard correction	/	0.10	0.1–50	89.8–97.2	1.3–9.3	[38]
UPLC-MS/MS	*Carassius auratus*, *Solenocera crassicornis*, *Portunus trituberculatus*, *Mytilus edulis*,	MSPE (Fe_3_O_4_@SiO_2_-PSA, C18)	Matrix-matched calibration curve	0.20	0.50	0.1–10	74.9–109	1.24–11.6	[39]
UPLC-MS/MS	*Larimichthys crocea*, *Trachypenaeus curvirostris*, *Carassius auratus*, *Portunus trituberculatus*, *Mytilus galloprovincialis*	MSPE (Fe_3_O_4_@SiO_2_-DVB-NVP, C18)	Solvent-based calibration curve	0.20	0.50	0.25–50	89.3–119.7	0.8–10.2	Present method

**Table 3:** Information on diazepam-positive samples.

Sample Name	Number of Samples	Detected Value (μg/kg)	Average Value(μg/kg)
*Mylopharyngodon piceus*	5	0.54–24.47	8.29
*Micropterus salmoides*	1	0.82	0.82
*Carassius auratus*	13	0.52–55.65	8.58
*Cyprinus carpio*	4	0.57–6.38	2.16
*Channa argus*	2	0.58–0.99	0.79
*Chanodichthys erythropterus*	3	1.31–1.76	1.51
*Aristichthys nobilis*	1	1.09	1.09
*Ctenopharyngodon idella*	1	0.55	0.55
*Acrossocheilus fasciatus*	1	1.01	1.01
*Opsariichthys bidens*	2	0.58–0.67	0.63
*Parabramis pekinensis*	2	0.65–1.46	1.06
*Piaractus brachypomus*	1	32.79	32.79
*Siniperca chuatsi*	1	1.95	1.95

## Data Availability

The original contributions presented in this study are included in the article. Further inquiries can be directed to the corresponding author.
